# *JAK2* Variant Signaling: Genetic, Hematologic and Immune Implication in Chronic Myeloproliferative Neoplasms

**DOI:** 10.3390/biom12020291

**Published:** 2022-02-11

**Authors:** Dania G. Torres, Jhemerson Paes, Allyson G. da Costa, Adriana Malheiro, George V. Silva, Lucivana P. de Souza Mourão, Andréa M. Tarragô

**Affiliations:** 1 Programa de Pós-Graduação em Ciências Aplicadas à Hematologia, Universidade do Estado do Amazonas (UEA), Manaus 69850-000, AM, Brazil; dgutorr@gmail.com (D.G.T.); jhemersonpaes@gmail.com (J.P.); allyson.gui.costa@gmail.com (A.G.d.C.); malheiroadriana@yahoo.com (A.M.); georgevillarouco@hotmail.com (G.V.S.); 2 Programa de Pós-Graduação em Imunologia Básica e Aplicada, Universidade Federal do Amazonas (UFAM), Manaus 69067-005, AM, Brazil; 3 Fundação Oswaldo Cruz–Instituto Leônidas e Maria Deane (Fiocruz), Manaus 69027-070, AM, Brazil; 4 Fundação Centro de Controle de Oncologia do Amazonas (FCECON), Manaus 69040-010, AM, Brazil; 5 Fundação Hospitalar de Hematologia e Hemoterapia do Amazonas (FHEMOAM), Manaus 69050-001, AM, Brazil

**Keywords:** *JAK2*V617F signaling, myeloproliferative neoplasms, clonal hematopoiesis, hemostasis, immunothrombosis, immune response

## Abstract

The *JAK2*V617F variant constitutes a genetic alteration of higher frequency in BCR/ABL1 negative chronic myeloproliferative neoplasms, which is caused by a substitution of a G ˃ T at position 1849 and results in the substitution of valine with phenylalanine at codon 617 of the polypeptide chain. Clinical, morphological and molecular genetic features define the diagnosis criteria of polycythemia vera, essential thrombocythemia and primary myelofibrosis. Currently, *JAK2*V617F is associated with clonal hematopoiesis, genomic instability, dysregulations in hemostasis and immune response. *JAK2*V617F clones induce an inflammatory immune response and lead to a process of immunothrombosis. Recent research has shown great interest in trying to understand the mechanisms associated with *JAK2*V617F signaling and activation of cellular and molecular responses that progressively contribute to the development of inflammatory and vascular conditions in association with chronic myeloproliferative neoplasms. Thus, the aim of this review is to describe the main genetic, hematological and immunological findings that are linked to JAK2 variant signaling in chronic myeloproliferative neoplasms.

## 1. Introduction

Chronic myeloproliferative neoplasms (MPNs) are clonal pathologies of hematopoietic stem cells [1], which are characterized by medullar hyperplasia and an accumulation of elements of the myeloid series, and present progressive and effective maturation [2], though without affecting the maturation and differentiation process of the erythroid, granulocytic and megakaryocytic lineage [3]. These changes lead to peripheral blood leukocytosis, increased erythrocyte mass, thrombocytosis and, in more severe cases, medullary fibrosis or leukemic transformation [2].

The first studies on MPNs date from 1845, starting with the description of the first case of chronic myeloid leukemia [4]. Since then, several scholars have engaged in the analysis of the molecular mechanism of chronic myeloproliferative neoplasms, and have determined the semiological aspects of these hematological diseases by observing the signs, symptoms and clinical findings of the investigated patients [5,6,7].

**Figure 1:**
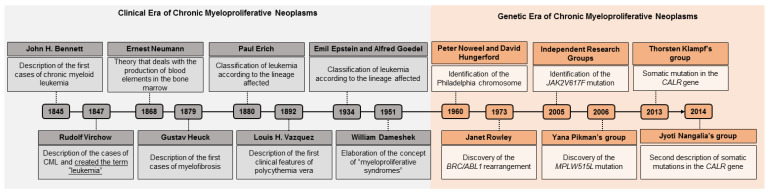
Timeline of myeloproliferative neoplasms. In gray, the clinical era of the MPNs can be seen, which is based on semiological aspects. In orange, the genetic era of MPNs is highlighted, since associated genetic research made it possible to identify genetic alterations that are markers of some MPNs, an important factor that supports diagnosis. CML: chronic myeloid leukemia; BCR/ABL: BCR/ABL genetic rearrangement.

This clinical view gained a new ally in 1960 with the discovery of the Philadelphia chromosome, which was the first association between a chromosomal abnormality and an oncological disease to be described in the history of medicine [3,4,8,9]. Since this discovery, the history of MPNs shows them to enter the era of genetics, during which more studies began to be developed and genetic research gained further space in the diagnosis of MPNs (Figure 1).

Thus, with the discoveries of molecular mechanisms involved in MPNs, the World Health Organization (WHO) determined clinical and laboratory parameters for establishing the diagnosis of these diseases and the WHO, according to its last review in 2016, classifies chronic myeloid neoplasms according to the presence or absence of the Philadelphia chromosome (BCR/ABL1 fusion gene) [10,11]. In the BCR/ABL1 positive MPN classification, chronic myeloid leukemia (CML) is responsible for 15–20% of leukemias worldwide, with an incidence of 1–2/100,000. CML is characterized by the presence of a left shift in granulocytes that results in the identification of less than 20% of the blasts in peripheral blood [4,10].

Unlike CML (single BCR/ABL1 positive entity, according to the WHO), diseases belonging to the group of BCR/ABL1 negative MPNs have been described, including polycythemia vera (PV), essential thrombocythemia (ET), primary myelofibrosis (PMF), chronic neutrophilic leukemia (CNL), chronic eosinophilic leukemia (CEL) and unclassifiable myeloproliferative neoplasms [10,11]. However, polycythemia vera, essential thrombocythemia and primary myelofibrosis are the most frequent diseases in this group [10,12,13], with PMF having the worst prognosis of the three myeloproliferative disorders [12,14]. Similar to CML, in the BCR/ABL1 negative MPNs, criteria are established based on genetic, hematologic and clinical findings, which are organized in the form of major and minor criteria for the diagnosis of PV, ET and PMF, as shown in Table 1.

**Figure 2:**
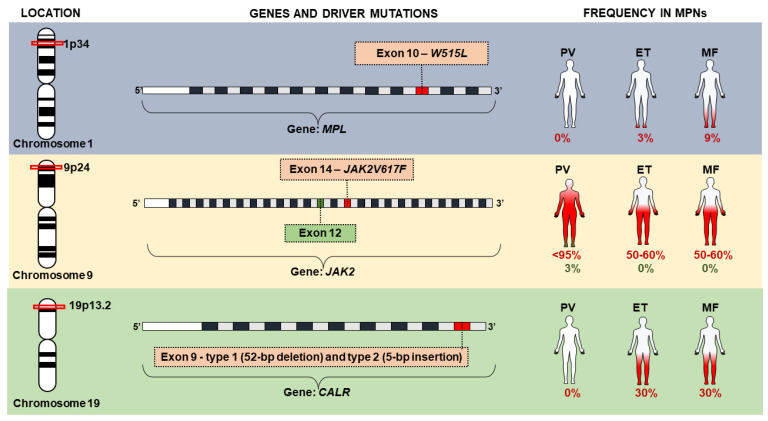
Driver mutations in *JAK2*, *MPL* and *CALR* genes associated with chronic myeloproliferative neoplasms (MPNs).

Among the major criteria of BCR/ABL1 negative MPN, the presence of variants in driver genes stands out, as well as the Janus kinase 2 gene (*JAK2*—HGNC: 6192), the thrombopoietin receptor gene (*MPL*—HGNC: 7217) and the calreticulin gene (*CALR*—HGNC: 1455). Variations in these genes, classified as driver mutations, are determinant in the clinical phenotype observed in MPNs and result in constitutive activation of intracellular signaling pathways [12,19] (Figure 2). Generally, these genomic variations are considered mutually exclusive between BCR/ABL1 negative entities, and their absence does not exclude their diagnosis [10]. However, two variants can be found in the same individual, probably from different neoplastic subclones and those that are often associated with disease progression [19,21].

These driver mutations usually arise again; however, 7% of cases involve familial aggregation, with autosomal dominant inheritance and incomplete penetrance, and a 5 to 7-fold increased risk for first-degree relatives of the patient with the disease, a risk that may involve the same or different myeloproliferative neoplasms [22,23,24]. Although *JAK2*V617F is linked to autosomal dominant inheritance, most cases of familial inheritance are not associated with *JAK2* gene variants [25]. Nonetheless, mutations in the *EPOR*, *VHL*, *EPAS1*, *HIF* and *EGLN* gene have been found in cases of congenital familial polycythemia [26].

Thus, some determining factors associated with driver mutations should be considered, such as mutations (somatic or germline) that are less specific for MPNs, gender, frequency of the allelic variant and order of acquisition of mutations [27]. In ET and PMF, 12% of patients do not have any of the driver mutations and are called triple-negative. However, whole-exome sequencing analyses have identified mutations in the *JAK2* and *MPL* genes that have a high rate of leukemic transformation in these patients [28].

Among the driver mutations, the *JAK2*V617F variant is the most frequent in triggering PV, ET and PMF [10], which are considered to be a major criterion in polycythemia vera, essential thrombocythemia and primary myelofibrosis [5,29,30,31]. The *JAK2*V617F variant presents a frequency that is greater than 95% in individuals with PV, and frequency is from 55% to 65% in individuals with ET and PMF, respectively [32] The presence of *JAK2*V617F causes aberrant signaling of the JAK/STAT pathway, which is an intracellular pathway that is involved in several biological processes, such as hematopoiesis, immune response and activation of other intracellular signaling pathways. Thus, the understanding of *JAK2*V617F signaling mechanisms and the main cells involved in immunothrombosis may provide a basis for the development of immunotherapeutic strategies in myeloproliferative disorders.

The constitutive signaling of *JAK2*V617F is linked to high expression of molecules that are related to the inflammatory response, immune dysregulation and manifestation of inflammatory states [33,34,35], which is a finding that currently constitutes research targets. Cytokines related to natural immunity are the most expressed in chronic myeloproliferative neoplasms, and are detected even in the medullary stroma [34]. This suggests that inflammation is related to bone marrow stromal initiation, which promotes medullary fibrosis and clonal expansion [35]. In peripheral blood, the interaction between *JAK2*V617F positive hematopoietic cells, endothelium and immunological molecules enhances the immunothrombosis mechanism, thus constituting an independent and unfavorable prognostic factor in the survival of patients with MPNs [36].

As such, the understanding of the *JAK2*V617F signaling mechanisms and the main cells involved in immunothrombosis may provide a basis for the development of immunotherapeutic strategies in myeloproliferative disorders. Therefore, in this review, we describe the *JAK2*V617F variant and its implications for genomic instability and immune dysregulation, as well as its relationship to the onset of chronic inflammation through cellular mechanisms.

## 2. *JAK2*V617F: Genetic Implication in Signaling Pathways

**Figure 3:**
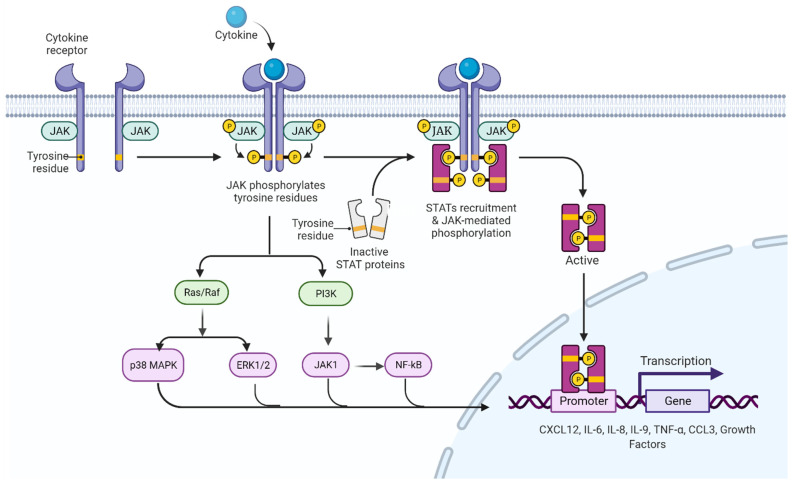
*JAK/STAT* signaling pathway. In the absence of cytokines, the JAK protein remains inactive in regions close to the intracellular domains of the receptor. When a cytokine binds to a receptor, *JAK* proteins and intracellular domains of the receptor are phosphorylated, activating and recruiting STAT proteins, which dimerize and translocate to the nucleus to initiate the transcription process of genes involved in cell proliferation. In addition to the activation of the JAK/STAT pathway, there is an interconnection with other intracellular signaling pathways, among the most prominent are the Ras/Raf/MAPK pathway and the PI3K pathway; the latter manifesting interconnection with JAK1 proteins, which indirectly activate the NFkB pathway, the transcriptional factors that activate the production of cytokines (among them CXCL12, IL-6, IL-8, IL-9, TNF-α and CCL3) and the growth factors identified in the inflammatory profile of individuals with chronic myeloproliferative neoplasms. *JAK*: Janus kinase protein; STAT: signal transducers and activators of transcription; MAPK: mitogen-activated protein kinase; PI3K: phosphoinositol kinase 3; JAK1: Janus kinase class 1 protein; NFkB: light chain nuclear factor B-cell kappa; CXCL12: chemokine 12 with CXC motif; IL-6: interleukin 6; IL-8: interleukin 8; IL-9: interleukin 9; TNF-α: tumor necrosis factor alpha; CCL3: chemokine ligand 3.

In 2005, the molecular basis of chronic myeloproliferative diseases was described, with the *JAK2*V617F variant (dbSNP ID: rs77375493) characterized by a transversion-type base substitution at nucleotide 1849 (1849G > T) of exon 14 of the *JAK2* gene encoding a valine through a phenylalanine at position 617 (V617F) [37,38,39]. Metabolically, this genetic alteration leads to a gain-of-function mutation in JAK2, a cytoplasmic tyrosine kinase with a central role in the signal transduction of hematopoietic growth factor receptors [40,41]. This change occurs in the pseudokinase domain of JAK2, interrupting the auto-inhibitory effect, and resulting in constitutive phosphorylation, which generates hyper phosphorylation, deregulates cellular signals downstream of the JAK2/STAT5 signaling pathway (Figure 3), interferes with the correct signaling of erythropoietin (EPO) receptors, granulocyte colony stimulating factor (G-CSF), granulocyte–monocyte colony stimulating factor (GM-CSF) and thrombopoietin (TPO), and also increases cell proliferation and resistance to apoptosis [ClinVar ID: NM_004972.3 (*JAK2*): c.1849G > T (p. V617F))] [3,41,42].

**Figure 4:**
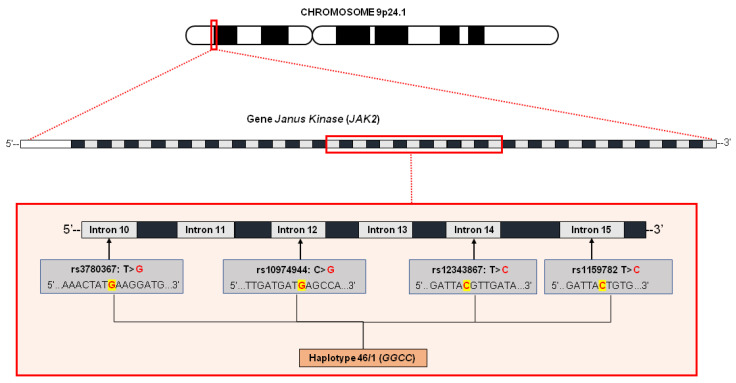
Characterization of the 46/1 haplotype without *JAK2* gene. The haplotype, also called GGCC, is altered by four variants located in intron 10 (rs3780367: T > G), intron 12 (rs10974944: C > G), intron 14 (rs12343867: T > C) and intron 15 (rs1159782: T > C). It is believed that the presence of this haplotype conditions an increase in the mutation rate of the gene locus in question, thus resulting in the emergence of mutations with a selective advantage, as in the case of *JAK2*V617F.

Analysis of germline cells, such as buccal cells, T cells or both, in cases of familial clustering of myeloproliferative disorders, show absence of the variant allele. In this scenario, the *JAK2*V617F variant is not the first event that leads to disease [22,40,43]. The 46/1 haplotype is a 280 Kb long region of chromosome 9p that includes three genes, including *JAK2*. The part called “GGCC” corresponds to the four main polymorphisms of this haplotype (rs3780367, rs10974944, rs12343867 and rs1159782) that start in intron 10 and end in intron 15 of the *JAK2* gene [44,45] (Figure 4). These four variants are in complete linkage disequilibrium, and are inherited together [44,46]. This haplotype is described as one of those responsible for the processes that precede the acquisition of *JAK2*V617F, increasing the mutation rate of the *JAK2* locus and the probability of acquiring mutations with selective advantage, which is the case of *JAK2*V617F and which, in turn, causes clonal myeloproliferative disorders [31,45,46,47,48].

It is still unclear how the same mutation is associated with three different disease phenotypes. Possible explanations include inter-individual differences in genetic background, acquisition of additional genetic alterations or in the target cell for transformation [43]. Thus, *JAK2*V617F may even compromise the functionality of cell lines that integrate the hematopoietic and inflammatory processes.

## 3. Implications of the *JAK2*V617F Variant in Positive Cells and Immunothrombosis

Recent studies have described the relationship between the mechanisms and immune responses expressed by cells involved in the innate and adaptive immune system (neutrophils, monocytes, macrophages, lymphocytes, endothelial cells and platelets) with important molecules of hemostasis, which is a phenomenon currently called immunothrombosis. The immunothrombosis process is multifactorial, generally mediated by hypercellularity, and causes changes in plasma proteins that are important in the process of hemostasis and activation of endothelial molecules, adhesion product and cell function [49,50,51]. Hypercellularity in peripheral blood is a result of the constitutive activation of the JAK2/STAT5 pathway, which increases blood viscosity through cell–cell–endothelial interaction, and even forms plasma complexes [49,50,51,52,53]. Thus, the characteristic leukocytosis, erythrocytosis and thrombocytosis in patients with MPNs not only reflect quantitative alterations in hematopoiesis, but also qualitative alterations in the immune response and hemostasis, through the expression of molecules that favor the activated prothrombotic phenotype [50]. It has been well described in the literature that vascular complications of arterial or venous type affect up to one third of individuals with MPNs and constitute one of the main causes of mortality in these individuals [51,52,54,55,56], especially in ET [53].

JAK/STAT pathway activation is involved in the inflammatory response by directly interconnecting with other intracellular signaling pathways involved in cytokine production. Di Rosa et al. [57] demonstrated that CD34+ cells from individuals with PMF showed dysregulated activation of the JAK2/STAT1 pathway and significant activation of genes involved in the IFN-γ pathway (*IFN-γ*, *IRF1* and *IFNGR2*) compared to healthy individuals, and noted that IFN I and II have been described as mediators of antitumor immunity through activation of the PI3K/AKT/mTOR pathway, which, in turn, activates the NFkB pathway and promotes antigen presentation and cytokine secretion [56].

On the other hand, the action of programmed death protein (PD-1), mediated by IFN-γ expression, which is vital in the tumor recognition process, is dysregulated in patients with MPNs, indicating that *JAK2*V617F positive hematopoietic cells from individuals with MPNs express the PD-L1 ligand, which is a mechanism that blocks the action of Th lymphocytes and contributes to the immune escape of neoplastic cells [58,59]. At the same time, this results in JAK2/STAT3 signaling up to three times greater than normal, which is a fact that is linked to tumor progression associated with inflammation [55]. The experimental murine assay carried out by Prestipino et al. [60] demonstrated oncogenic activity of JAK2 with consequent phosphorylation of STAT3 and STAT5 that facilitates the promotion of PD-L1 activity in *JAK2*V617F positive cells and affects the cell progression cycle of T cells, which is a finding associated with more advanced states of MPNs.

In PMF, there is an altered regulation of T cells, which is determined by the significant activation of CD8+ T lymphocytes, and this finding is linked to the activation of HLA class I molecules, chronic inflammation and immune dysregulation, thus favoring the activation of fibroblasts and contributing to the progression of medullary fibrosis and cytopenias [54]. Interestingly, the constitutive activation of *JAK2*V617F not only produces alterations in the cellular immune response through classical pathways, it also favors the indirect activation of hypoxia inducible factors (HIF) and erythropoietin (EPO) secretion through the NFkB pathway, generating an hypoxic state of variable severity that favors tissue atrophy and production of erythroid progenitors, an event that contributes to the production of pro-inflammatory cytokines in peripheral blood [58].

Therefore, *JAK2*V617F complexly deregulates intercellular signaling through activation of cytokine production and interruption of homeostasis and cytotoxicity of immune cells [48]. Immune dysregulation in individuals with MPNs is confirmed by high concentrations of IL-6, IL-8, GM-CSF, HGF, VEGF, b-FGF and TGF-β in medullary stromal cells and increased production of IL-6, IL-8, IL-9, CCL3, CCL4 and TNF-α in peripheral blood cells [59].

**Figure 5:**
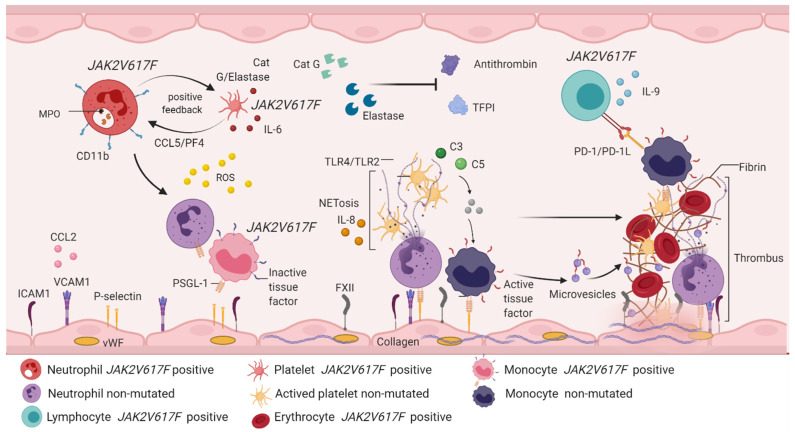
Immunothrombosis involves the participation of hematopoietic cells and immune system cells. *JAK2*V617F positive neutrophils express CD11b on the cell surface and secrete enzymes such as MPO, cathepsin G and elastase. The release of cathepsin G and elastase activates CCL5/PF4 signaling in *JAK2*V617F positive platelets, which is an interaction that creates positive feedback between platelets and neutrophils, and favors the production of IL-6 and ROS. Free cathepsin and elastase inhibit the function of antithrombin and plasma TFPI, contributing to the thrombotic phenotype. Unmutated neutrophils are activated by the action of ROS, which recruit and activate monocytes with PSGL-1 expression and inactive tissue factor. Both cells manifest rolling and endothelial adhesion by binding adhesion molecules expressed in endothelial cells (ICAM1, VCAM1, P-selectin, CCL2 and FXII). Simultaneously, endothelial cells are activated by the action of ROS, releasing vWF and collagen, thus forming a platelet–monocyte–neutrophil interaction, which favors the discharge of genetic and protein material from the neutrophil, starting with NETosis. This brings with it the expression of IL-8 and activation of TLR4/TLR2 in platelets, a fact that allows the expression of receptor glycoproteins and, therefore, platelet aggregation. In NETosis, erythrocytes are recruited and release hemoglobin and increase ROS production. The complement system is active in response to activation of immune cells, especially through the action of C3 and C5, which induces signaling from *JAK2*V617F positive monocytes and non-mutated monocytes. *JAK2*V617F positive monocytes express PD-1L, which immediately activates PD-1 in *JAK2*V617F lymphocytes, a mechanism responsible for the evasion of the immune response described in chronic myeloproliferative neoplasms. Recruitment and activation of immune and hematopoietic cells benefits the conversion of fibrinogen into fibrin, giving rise to thrombus formation, the main factor involved in vascular complications described in myeloproliferative neoplasms. MPO: myeloperoxidase; CCL5: chemokine ligand 5; PF4: platelet factor 4; IL-6: interleukin 6; ROS: reactive oxygen species; TFPI: plasma tissue factor inhibitor; PSGL-1: P-selectin ligand 1; ICAM-1: intercellular adhesion molecules 1; VCAM-1: vascular cell adhesion molecule 1; CCL2: chemokine ligand 2; FXII: factor XII; vWF: Von Willebrand factor; IL-8: interleukin 8; TLR2/TLR4: Toll-like receptor 2/4; C3: complement component 3; C5: complement component 5; PD-1: programmed death protein; PD-L1: programmed death protein ligand 1.

The inflammatory picture is related to the excess of cytokines (generally type I IFN and belonging to the IL-6 family), a product of the constitutive signaling of the JAK2/STAT5/STAT3 pathway, which directly activates other intracellular signaling pathways committed to the production of pro-inflammatory cytokines [28]. PI3K, MAPK, NFkB and HIF1-α pathways induce tumorigenesis [31] and expression of mediator mechanisms of inflammation in neutrophils, monocytes, macrophages, lymphocytes and platelets [60] (Figure 5).

### 3.1. Neutrophils

Neutrophils constitute a large percentage of leukocytes in peripheral blood and are involved in immune response processes against antigens. Neutrophils from individuals with MPNs have abundant basal amounts of reactive oxygen species (ROS), especially in patients with PMF, which are induced by JAK2-dependent ERK signaling and constitutive phosphorylation, producing NADPH oxidase and neutrophil activation by myeloperoxidase expression [61].

The activation and recruitment of leukocytes contributes to the formation of neutrophil extracellular traps (NETs), which are networks made up of genetic material and protein derived from neutrophils, and which favor cell activation, production of reactive oxygen species, platelet activation and aggregation and endothelial damage [62]. Activated neutrophils express CD11b on the cell surface and secrete elastase and myeloperoxidase, which favors the chemotaxis of neutrophils, monocytes and macrophages and facilitates endothelial adhesion [63,64,65,66]. Simultaneously, the expression of β1 and β2 integrins in recruited neutrophils is favored, and glycoproteins are expressed by interconnection of the JAK/STAT pathway with Rap1-GT-Pasa, which have affinity for vascular cell adhesion molecules (VCAM-1) and for intercellular adhesion molecules (ICAM-1) expressed by endothelial cells [67,68,69,70], thus enhancing endothelial adhesion. Likewise, expression of P-selectin ligand 1 (PSGL-1) and cell adhesion molecule type 1 (MAC-1) in neutrophils contributes to the release of cathepsin G (a neutrophilic degradative enzyme important in the elimination of pathogens and in the degradation of components in inflammatory sites), which, together with elastase, determines a positive feedback through the expression of CCL5 and platelet factor 4 (PF4) in platelets, stimulating the expression of P-selectin and GPIba in platelets and favoring the thrombogenesis process [52].

An interesting finding is that both elastase and cathepsin G block the tissue factor pathway inhibitor (TFPI) and antithrombin (AT), which are two potent natural anticoagulants, and this contributes to the activation of the proteinase 4 receptor (PAR4) pathway in platelets, Von Willebrand factor exposure and initiation of the coagulation cascade [51,71].

Thus, neutrophil activation allows the release of DNA-histone complexes (especially H3 and H4) that induce the production of NETs and platelet activation via NF-kB and TLR2 and TLR4 function [67], with a consequent expression of GPaIIb3 contributing to platelet aggregation and formation of thrombin in the extrinsic pathway [68]. Histone-MPO complexes have also been found in the plasma of individuals with MPNs and to be associated with high levels of LDH [61]. As such, NETosis plays a crucial role in tumor expansion in MPNs by enhancing immunothrombosis and activating hemostasis, thus forming a repetitive cycle [66].

Since we now know about the active participation of neutrophils in the process of immunothrombosis in MPNs, there has been great interest in identifying neutrophil subtypes involved in this mechanism. Tumor-associated neutrophils (TANs), also classified as PMN-MDSCs, can be subdivided into N1 (neutrophils with anti-tumor action) and N2 (neutrophils with pro-tumor action) [68,72,73], both in human and murine models, and are found in circulation and in the microenvironment of patients with tumorigenic and inflammatory conditions [70]. TANs directly contribute to the angiogenesis process through significant release of ROS and consequent formation of NETs through expression of CD11b and elastase [71].

The presence of TANs is also documented in processes of infiltration, invasion and metastasis of solid tumors, such as melanoma, advanced gastric carcinoma, infantile brain tumor [72] and even in cases of acute pancreatitis [74,75,76,77]. In these types of tumors, TANs are sensitive to microenvironmental signals caused by the secretion of CXCL1, CXCL2, TNF-α, IFN-γ and IL-8 [70], which favors local invasion, functional overexpression of neutrophils and functional suppression of T lymphocytes. However, the role of TANs in hematologic malignancies has been sparsely described. In 2019, the study by Podaza and Risnik [78] aimed to detect TANs in individuals with chronic lymphocytic leukemia (CLL) and found that the proportion of TANs in patients with CLL was greater, with high concentrations of IL-8, an important molecule in the induction of NETs. Unfortunately, due to the scarce description of surface markers in this classification of neutrophils, the research does not discriminate the TAN (N1∕N2) phenotypes found, which are defined by the production capacity of ROS and NETs. Nonetheless, some recent studies have aimed to describe the phenotyping of these neutrophil subpopulations to better characterize them, especially their role in hematological diseases.

### 3.2. Platelets

Platelet activation and dysfunctionality is a well-described phenomenon in thrombotic and hemorrhagic processes in patients with MPNs, and it is worth mentioning that some platelet markers, such as P-selectin, CD41L, β-thromboglobulin, PF4 and platelet-derived growth factor (PDFG), have been detected in high concentrations in the plasma of individuals with ET and PV [52,54], which are biomarkers associated with thrombotic complications. The expression of these markers induces a hemostatic response through exposure and activation of tissue factor (a product of endothelial damage), directly activating the extrinsic pathway of the coagulation cascade and consequent fibrin production. In this process, activated platelets expose negatively charged phospholipids that confer proteolytic reaction of coagulation factors, acting as mediators between coagulation and inflammation through activation of the complement cascade, especially of C3 and C5 molecules [66].

Interestingly, patients with MPNs can also manifest high basal levels of phosphatidylserine in the platelet membrane, which in turn develops a pro-coagulant function, and which is a finding that demonstrates that platelets are the central target in the development of vascular and inflammatory complications in MPNs [75]. Thus, the presence of the *JAK2*V617F mutation could potentiate platelet activation and contribute to persistent thrombosis in these patients.

On the other hand, the constant circulation of complexes and activation of platelet proteins can expose molecules derived from endothelial cells, favoring endothelial activation and damage through TLR-4 signaling in platelets [76]. As such, endothelial activation is promoted by high levels of reactive oxygen species, which are released by neoplastic platelet–neutrophil complexes that lead to exposure of Von Willebrand factor, collagen, platelet–endothelial cell adhesion molecules (PECAM), E-selectins and thrombomodulin, as well as CD40L (an important tissue factor inducer), which are all molecules that favor endothelial adhesion and are directly involved in the activated thrombotic picture [75,79]. In the active process of hemostasis, the expression of some biomarkers, such as thrombin, D-D, FVIII, fibrinogen, CD40L and platelet integrins, is detected; that, together with tissue factor, protein S, protein C, Von Willebrand factor and P-selectins, favors the perpetuation of active hemostasis, cell adhesion and recruitment of leukocytes and erythrocytes [76]. Although platelets form complexes with neutrophils, platelet–monocyte aggregates have also been found, especially in patients with ET [77].

The interaction between platelets and endothelial cells most likely contributes to the production of soluble selectins and the reduction of nitric oxide (a consequence of high levels of reactive oxygen species and MPO), which benefits the laminar vessel reduction and vascular obstruction [51,80]. Poisson et al. [81] provide confirmation of this, in which endothelial cells showed a dysfunction of the nitric oxide pathway, the cause of the accumulation of microvesicles derived from MPO-carrying erythrocytes, which increased endothelial oxidative stress and compromised the vascular response to vasoconstrictors. This implies a possible participation of erythrocytes in this process. Therefore, in hematopoietic cells, oxidative stress and chronic inflammation status is favored by the vicious cycle of biomacromolecule production that contributes to genomic instability, mutation acquisition, tissue damage and acute leukemia transformation [82,83].

On the other hand, hemorrhagic conditions can also be presented in patients with MPNs, and are linked to increased platelet consumption (product of thrombogenesis) due to the absence of a connection with high molecular weight multimers of the Von Willebrand factor and dysfunctionality of platelet-dense granules [84]. However, hemorrhagic complications are more often described in individuals with PMF and PV, compared to those with ET [85]. An important finding about this is that individuals with mutated ASXL1-PMF have a poor prognosis and a high risk of complications in hemostasis [86]. Unfortunately, a relationship between mutations in *ASXL1* and the development of bleeding disorders in patients with PMF has not yet been established.

Matsuura et al. [87] observed a significant reduction of platelet-dense granules in *JAK2*V617F positive platelets, suggesting that hyperactivation in JAK2 affects the development of an ensemble of these granules in platelets in the thrombopoiesis process. As a result, the role that JAK2 signaling plays in the control of dense granules in platelets has never been reported.

### 3.3. Monocytes

Monocytes play an important role in the process of immunothrombosis, which is also an independent and unfavorable prognostic factor in the survival of patients with PV and PMF, since they are mediators of inflammation, thrombosis and medullary fibrosis due to the secretion of high concentrations of cytokines and the presentation of an unbalanced response to IL-10. In the bone marrow, the pro-inflammatory cytokine signaling promotes the interaction between malignant clone and stromal cells, which stimulates osteoclastogenesis in the endosteal niche and causes the emergence of fibrocyte clones involved in the induction of medullary fibrosis in PMF [36]. This mechanism is promoted through cell cycle dysregulation in fibroblasts, and accelerates the mal-differentiation process so that it loses the ability to repair hematopoietic tissue (may be a product of the oxidative stress) and contributes to marrow fibrosis [88].

By using single-cell RNA-seq, Leimkuhler et al. [89] demonstrated that transcriptomics of mesenchymal stromal cells of primary myelofibrosis patients show loss of hematopoietic niche support, decreased multipotent progenitor status cell, upregulated JAK/STAT and TGF-β signaling and upregulation of extracellular matrix proteins like collagen.

Indirectly, monocyte activation is determined by exposure to pathogen-associated molecular patterns (PAMPs) and damage-associated molecular patterns (DAMPs), thus providing exposure to tissue factor and CD25 and benefiting activation of proteins with procoagulant action and NFkB signaling [90].

On the other hand, monocyte activation may be favored by the expression and binding of PSGL-1 to platelet P-selectin, favoring the expression of inflammatory cytokines [71]. Therefore, platelet–monocyte interaction induces a pro-inflammatory phenotype through the expression of CD147, PSGL-1, EP1/EP2 and COX-2 as well as the activation of integrins that contribute to endothelial adhesion and monocyte recruitment [91]. Confirmation of this is found in the study by Wei Wang et al. [52], in which *JAK2*V617F positive macrophages manifested a high expression of inflammatory cytokines (IL-1β, IL-6 and TNF-α), nitric oxide synthase (iNOS), ligand-2 chemokine (CCL2) and activation of the *MAPK* pathway, and they noted that monocytes showed remarkably distinct rolling and cell adhesion when compared to wild-type cells.

It is important to mention that macrophages are subdivided into type M1 and type M2, that both cell subtypes are activated by the NFkB signaling pathway, and that they have cellular functions similar to N1 and N2, respectively [92]. M2 is the largest component in neoplastic tissues, and directly contributes to the tumor environment, proliferation, angiogenesis and release of cytokines that lead to neoplastic expansion [93]. However, M2 has been sub-classified into M2a, M2b and M2c; though it is worth noting that monocyte/macrophage accumulation of M2b can promote growth, invasion and recurrence of cancers in vitro and in vivo [93].

In the study by Molitor et al. [94], it was observed that *JAK2*V617F positive M2 in patients with PMF showed greater capacity for colony formation through the secretion of pro-fibrotic molecules, such as CCL2, IL-8, matrix metalloproteinase-9 (MMP9), galectin 3 (LGALS3) and osteopontin (SPP1), which drive the proliferation and production of collagen. Nonetheless, further research is needed regarding the role of macrophage subclasses in different MPN phenotypes.

### 3.4. T Helper and Natural Killer Lymphocytes

Although lymphocytes are not well described in MPNs, these cells may also be involved in immune dysregulation. T lymphocytes have a long lifespan and this possibly contributes to greater signaling of disruptive effects in the immune system [95]. In some PMF cases, T lymphocytes carry 3–83% of variant allele frequency of *JAK2*V617F, accompanied by abnormalities in the karyotype, such as 13q-, 20q-, monosomy 7 and inv(3). It is noted that these last two chromosomal alterations confer an unfavorable prognosis [95,96,97,98]. Just as in lymphocytes, the role of natural killer (NK) cells in MPNs has not been widely described.

NK cells are associated with the control of tumor growth and metastasis, which indicates that NK cell deficiency is very rare and is generally linked to lymphoproliferative disorders [98]. However, the study by Arantes [99] demonstrated that NK cell *JAK2*V617F-positive patients had lower NKCD16 + CD56^dim^ counts compared to NK cell *JAK2*V617F-negative patients, especially those with PV and PMF, which is a finding that suggests that NK cells may be defective in MPNs. Although there have been some investigations into the role of lymphocytes and NK cells in these hematological diseases, further research is needed in order to elucidate their role in the evolution of these diseases and in the development of immunothrombosis in MPNs.

## 4. Summary and Perspectives

The clinical approach to patients with myeloproliferative neoplasms is based on the control of vascular complications, both arterial and venous, since these are events that result in the main cause of morbidity and mortality due to hematological diseases. According to recent investigations, thrombohemorrhagic complications are attributed to the presence and high allelic frequency of *JAK2*V617F [100], suggesting that the presence of the *JAK2* 46/1 haplotype has not been related to the production of inflammatory biomarkers that can be expressed in immunothrombosis [101]. Likewise, the link between *JAK2*V617F and dysregulations in the immune response and hemostasis is well-described and associated with the interconnection of the JAK/STAT pathway with other intracellular signaling pathways, such as PI3K/AKT, Ras/Raf/MAPK and NFkB, in the apoptotic process and in the production of inflammatory molecules [59]. In addition, next-generation sequencing investigations have described other genes involved in these signaling pathways (*FLT3, GNAS, KIT, KRAS, NF1, NRAS, PTPN11* and *SH2B3*), in epigenetic regulator genes (*TET2, ASXL1, DNMT3A* and *EZH2*) and negative regulator genes of the JAK2 signaling pathway (*SOCS* and *CBL*), since they showed a strong association with dysregulations in cytokine production and secretion [102,103]. These could be excellent research targets in pathogenic mechanisms of MPN, and be employed as predictors of worse clinical outcome, stratification risk or even as leukemia transformation predictors [104].

The International Working Group—Myeloproliferative Neoplasms Research and Treatment (IWG–MRT) established a prognostic algorithm for thrombotic complications in common with *BCR/ABL1* negative chronic myeloproliferative neoplasms, especially in PV and ET, based on the variables of age (>60 years), percentage of homozygosity of *JAK2*V617F and history of thrombotic events, which are factors that define the categories of low risk, very low risk, intermediate and high risk of thrombotic and vascular complications [5].

Cytoreductive therapy in individuals with PV, ET and PMF is often scrutinized but widely used to alleviate the clinical picture and reduce the risk of these complications. Thus, the drug of choice in individuals with PV and ET is hydroxyurea (HU). Unfortunately, resistance or intolerance to HU have been described in 15–20% of patients with MPN [105]. This phenomenon is a challenge in the treatment of these patients because it reduces the therapeutic options and increases disease progression or the thrombotic risk [105]. Many investigations regarding this have been carried out and suggest that the existence of other molecular alterations in the kinase domain of JAK2 protein or in non-driver genes could be the reason for the pharmacological refractory [106,107].

Ruxolitinib, a drug approved a few years ago by the FDA, is also considered an excellent pharmacological choice in patients resistant to HU in PV [108]. However, murine models demonstrated that its pharmacological action does not present anti-leukemic activity in vivo in the bone marrow [109], which is a finding that could suggest a possible contribution from other deregulated cellular signaling, and is a target that would also be interesting in the prognosis of these patients. In PMF, the treatment of choice is HU, and using ruxolitinib for cases refractory to HU and patients classified as high risk [109]. In young high-risk patients with PMF, allogeneic hematopoietic stem cell transplantation is considered. Nevertheless, the use of cytoreductive drugs for reticular fibrosis or collagen would increase the chances of survival in these individuals; it is worth noting that PMF is the *BCR/ABL1* negative chronic myeloproliferative neoplasm with the greatest association with leukemic transformation.

On the other hand, inhibition of platelet function in *BCR/ABL1* negative chronic myeloproliferative neoplasms is one of the main functions, since, as previously described, platelets play a central role in the process of immunothrombosis. Thus, the use of antiplatelet agents reduces the rate of occurrence and recurrence of thrombo-hemorrhagic and inflammatory events [80]. Low molecular weight heparin, aspirin and clopidogrel are the main drugs used to control platelet activation. Although they are the most frequently used drugs in these diseases, the use of new drugs that inhibit the expression of platelet receptors, platelet adhesion molecules and cytokines would favor the reduction of thrombus formation and, consequently, the development of immunothrombosis [5]. Likewise, inhibition of the expression of integrins, adhesion molecules and other membrane proteins in neutrophils, monocytes, lymphocytes and endothelial cells constitutes a promising strategy in individuals with *BCR/ABL1* negative chronic myeloproliferative neoplasms as treatment targets [66], especially in those with drug resistance and categorized as high risk.

Despite the detection of microvesicles, cellular complexes and other inflammatory markers have been comprehensively discussed in MPN, and detection of mi-RNAs is actually considered a metabolic response marker, before, during and after lymphoma treatment [110]. Moreover, the advance in molecular techniques leads us to the development of noninvasive techniques, such as liquid biopsy, that could be a potential tool, especially in non-solid cancers, for prognosis and monitoring indicators in hematologic malignancies [111]. Currently, analyzing the circulating cell-free (cf)-DNA is proposed as a noninvasive tool for use in the diagnosis and prognosis of hematologic malignancies, which could be employed to differentiate between MPN subtypes, as well as to predict the development of thrombotic complications [111,112].

## Figures and Tables

**Table 1:** Diagnosis criteria for classic BCR/ABL1 negative chronic myeloproliferative neoplasms.

Neoplasm	Clinical Description	Major Diagnostic Criteria	Minor Diagnostic Criteria
**PV**	Exacerbated increase in erythrocyte mass (total red blood cell count). Generally, both genders are diagnosed in the 6th or 7th decade of life [11]. Annual global incidence is 0.3–1.5/100,000 and survival rate is 15 years [2].	Hb: >16.5 g/dL for men (or Hct: >49%), >16.0 g/dL for women (or Hct: >48% women), or >25% increase in red cell mass [2]; Bone marrow biopsy demonstrates panmyelosis with pleomorphic mature megakaryocytes; Presence of the *JAK2*V617F mutation or mutations in exon 12 of the *JAK2* gene.	Reduced serum erythropoietin concentration [10].
**ET**	Increased platelet count with megakaryocytic hyperplasia. Annual global incidence is 1.03–2.5/100,000 and diagnosis usually occurs in the 6th decade of life [15,16]. Together with PV, it presents high risks of hemorrhagic and thrombotic episodes [3,12,17,18].	Platelet count ≥450 × 10^3^/mm^3^; Hyperproliferation of megakaryocytes (some of them hyperlobulated, and observed in bone marrow biopsy); Mild increase in granulopoiesis and erythropoiesis; Absence of criteria for PV, CML and PMF; Presence of mutations in the *JAK2* gene (*JAK2*V617F), *CARL*, *MPL.*	Presence of clonal marker or absence of evidence of reactive thrombocytosis [10].
**PMF**	Indolent clinical course and has worse prognosis. Patients show increased megakaryopoiesis and extramedullary hematopoiesis [2,3,19,20] It has an annual global incidence of 1.5–2.0/100,000, and generally affects individuals over 60 to 70 years of age.	Pre-fibrotic phase: Exacerbated proliferation of the megakaryocytic and granulocytic lineage, absence of reticulin fibrosis >1, decreased erythropoiesis and medullary hypercellularity for the patient’s age; Absence of criteria for CML, PV and ET; Presence of mutations in the *CALR* gene, *JAK2* (*JAK2*V617F) or *MPL* or other clonal marker.	Pre-fibrotic phase: Anemia with no known cause; Leukocytosis 11 × 10^3^/mm^3^ Palpable splenomegaly; Increased lactic dehydrogenase.
		Fibrotic phase: Megakaryocytic proliferation and atypia, accompanied by grade 2 or 3 reticulin and/or collagen fibrosis; Does not meet the criteria for PV, ET, CML, MDS or other myeloid neoplasms; Presence of mutations in the *CALR*, *JAK2* (*JAK2*V617F) or *MPL* gene or absence of reactive fibrosis.	Fibrotic phase: Anemia with no known cause; Leukocytosis 11 × 10^3^/mm^3^; Palpable splenomegaly; Increased lactic dehydrogenase Leukoerythroblastosis.

MPN: myeloproliferative neoplasms; PV: polycythemia vera; ET: essential thrombocythemia; PMF: primary myelofibrosis; Hb; hemoglobin; Ht: hematocrit; CML: chronic myeloid leukemia; MPL: thrombopoietin receptor gene; CALR: calreticulin gene.

## Data Availability

Not applicable.
